# Urinary 3-methylhistidine as a potential biomarker for sepsis-associated acute kidney injury: multidimensional metabolomics analysis in mice and human

**DOI:** 10.1186/s13613-025-01550-z

**Published:** 2025-08-26

**Authors:** Xibo Wang, Pengfei Huang, Yinghao Luo, Yu Xin, Yue Li, Lifeng Shen, Yanqi Liu, Yang Zhou, Yuxin Zhang, Qianqian Zhang, Dawei Wang, Feiyu Luan, Weiting Zhang, Mengyao Yuan, Yuhan Liu, Fengye Liu, Nan Zhang, Jinyuan Wu, Tao Wu, Xuan Wang, Yuping Bai, Mingyan Zhao, Changsong Wang, Kaijiang Yu

**Affiliations:** 1 Departments of Critical Care Medicine, The First Affiliated Hospital of Harbin Medical University, Harbin Medical University, Harbin, 150001 Heilongjiang China; 2 Department of Critical Care Medicine, The Second Affiliated Hospital of Harbin Medical University, Harbin, 150086 Heilongjiang Province China; 3 Department of Critical Care Medicine, Harbin Medical University Cancer Hospital, Harbin, 150081 China; 4 Heilongjiang Provincial Key Laboratory of Critical Care Medicine, Harbin, Heilongjiang Province China

**Keywords:** Sepsis-associated acute kidney injury, Sepsis, Metabolome, Urine, 3-Methylhistidine, Diagnostic biomarkers

## Abstract

**Background:**

Sepsis-associated acute kidney injury (SA-AKI) is strongly associated with increased mortality in critical patients. The early detection of SA-AKI is crucial for clinical intervention. This study aims to integrate multiple metabolomics data related to SA-AKI to identify and validate novel metabolic markers.

**Methods:**

Real-time glomerular filtration rate (RT-GFR) measurement was adopted to establish SA-AKI mice. Untargeted metabolomics sequencing was performed on SA-AKI mice renal tissue (Control—LPS-8 h—LPS-24 h, N = 4) and urine samples (Control group *vs.* LPS-24 h group, N = 6). Time series analysis and random forest algorithm were employed to identify key metabolic molecule. Subsequently, renal spatiotemporal metabolomics was used to explore the specific distribution of key molecule. Eventually, a clinical cohort (20 healthy volunteers *vs.* 30 sepsis patients *vs.* 45 SA-AKI patients) urine quantitative metabolomic analysis was carried out to validate it as a biomarker and construct a diagnostic model via logistic regression (LR).

**Results:**

Forty-two key renal metabolites and top fifty urinary metabolites were determined through multidimensional metabolomics study of SA-AKI mice. Urinary 3-Methylhistidine (3-MH) was charactered as a potential biomarker. The distribution of 3-MH increased in collecting ducts through renal spatiotemporal metabolomics sequencing. Then, we recruited 95 urine samples to validate its diagnostic performance (AUC = 0.86, 95% CI 0.77–0.95) and its role as an independent predictive factor for SA-AKI (OR = 0.21, 95% CI: 0.05–0.84,* p* < 0.05). Ultimately, a diagnostic model combined urinary 3-MH with clinical variables was constructed to identify SA-AKI (AUC = 0.89, 95% CI 0.74–1.00).

**Conclusions:**

We proposed that urinary 3-Methylhistidine has potential diagnostic value for SA-AKI screening. Future studies will focus on its performance in other clinical populations to comprehensively evaluate its diagnostic role.

**Supplementary Information:**

The online version contains supplementary material available at 10.1186/s13613-025-01550-z.

## Background

Sepsis is a life-threatening condition characterized by organ dysfunction resulting from a dysregulated host response to infection [1]. It is estimated that millions of people lose their lives annually due to sepsis and its complications [2, 3]. Acute kidney injury (AKI) is one of the most common complications of sepsis which significantly increase the risk death of patients with sepsis [4, 5].

Currently, the diagnosis of AKI is primarily dependent on serum creatinine (SCr) and urine output [6–8]. However, both these indicators might delay the diagnosis of AKI. SCr exhibits significant individual variability, resulting in insufficient sensitivity and specificity [9]. On the other hand, urine output is influenced by fluid volume, cardiac function, and medications, all of which limit its reliability as a diagnostic tool [10]. Several novel biomarkers such as Neutrophil Gelatinase-Associated Lipocalin (NGAL), Kidney Injury Molecule-1 (KIM-1), and Liver-Type Fatty Acid-Binding Protein (L-FABP) have been identified to AKI diagnostic recently [11–14]. Nevertheless, the efficiency of these markers is inadequate, and they do not show significant dynamic changes in the early stage of renal injury progression [15]. Moreover, they are not specific to sepsis-associated acute kidney injury (SA-AKI) [16]. Therefore, it is more urgent to explore highly sensitive and specific biomarkers for the early diagnosis of SA-AKI.

The innovation of high-throughput sequencing technologies such as metabolomics and proteomics has significantly enhanced the opportunity to discovery biomarkers [17–21]. Metabolite can amplify upstream gene or protein signal from the central rule, which making it more sensitive to phenotypic changes [22, 23]. For instance, Xu et al*.* found that a model combination of phenylalanyl-tryptophan and glycocholate in a high-risk population for hepatocellular carcinoma showed better diagnostic capability than alpha-fetoprotein (AFP) [24]. However, the spatial position information can be quite limited if we only rely on high performance liquid chromatography-mass spectrometry (HPLC–MS) or nuclear magnetic resonance (NMR) to detect these metabolites [25]. These sequencing methods do not provide insight into whether metabolic reprogramming induced by renal tissue injury has specific spatial distribution characteristics [26, 27]. Therefore, this study aims to identify a novel metabolite biomarker for SA-AKI via urinary and renal metabolomics, and the potential of diagnostic role for SA-AKI was evaluated through spatial renal metabolomics and quantitative urinary metabolomics in clinical practice.

## Methods

### Study design

**Fig. 1:**
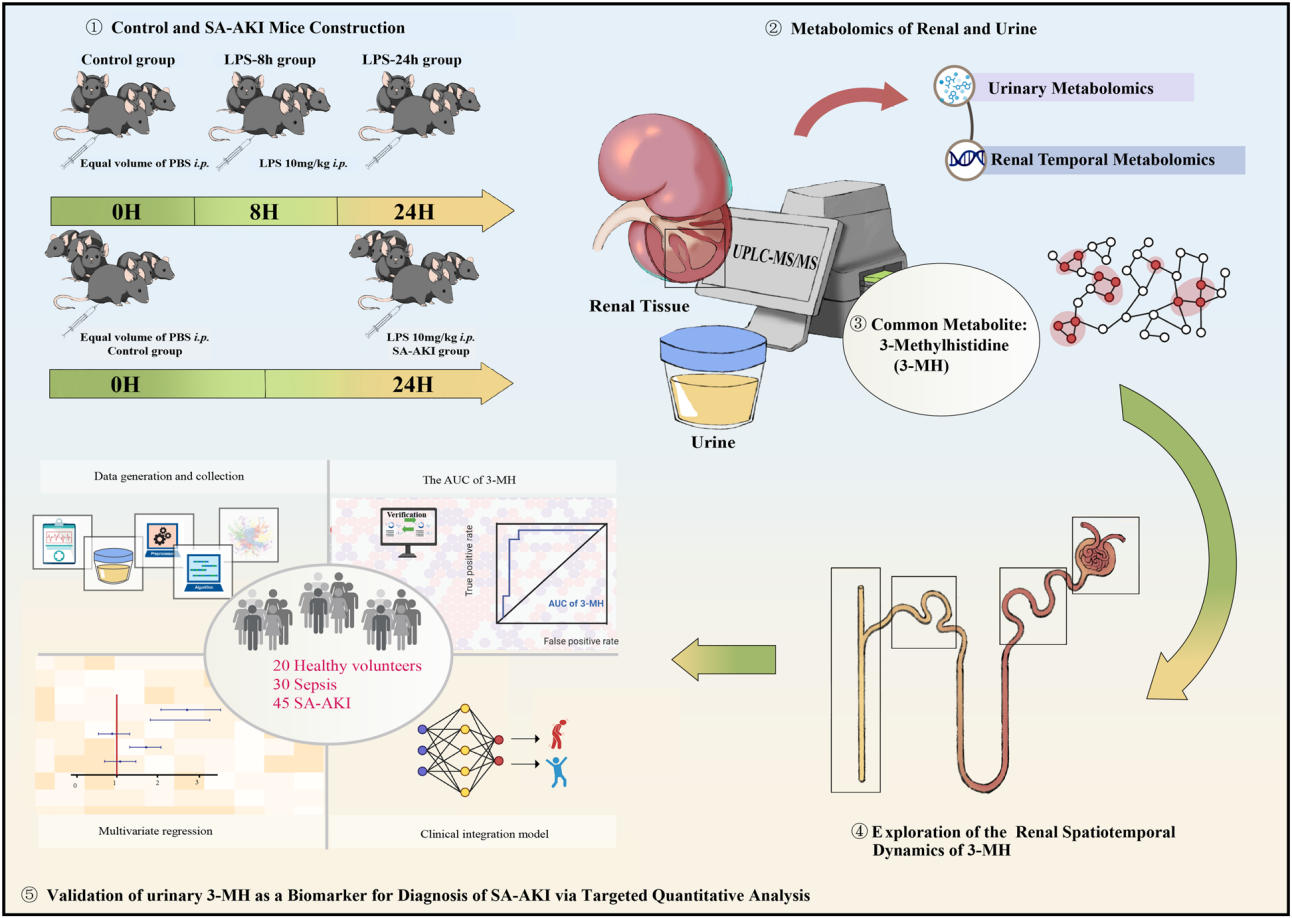
Research Design. Biotechnology, bioinformatics and machine learning algorithms were integrated in this study to identify urinary 3-Methylhistidine (3-MH) as a diagnostic biomarker for SA-AKI. Firstly, SA-AKI mice were established with glomerular filtration rate (GFR) measured via real-time fluorescence imaging. 3-MH was detected from renal temporal metabolomics (Control—LPS-8 h—LPS-24 h, N = 4) and urinary metabolomics (Control *vs.* LPS-24 h, N = 6). Subsequently, renal spatiotemporal metabolomics (Control—LPS-8 h—LPS-24 h, N = 4) was employed to investigate the specific spatial distribution of 3-MH. Finally, urinary 3-MH was recognized as a biomarker via clinical target quantitative metabolomics (healthy volunteers *vs.* sepsis patients *vs.* SA-AKI patients, N = 95) and a diagnostic model was constructed combining urinary 3-MH and clinical variables

We aimed to detect an early urinary biomarker for sepsis-associated acute kidney injury (SA-AKI) through a series of metabolomics and clinical data (Fig. 1). Firstly, 3-Methylhistidine (3-MH) was identified as a diagnostic role via integrating lipopolysaccharide (LPS)-induced SA-AKI mice renal time series metabolomics (Control—LPS-8 h—LPS-24 h, N = 4) and urinary metabolomics (Control group *vs.* LPS-24 h group, N = 6). Then, the potential of 3-MH as early biomarker was evaluated through mice renal spatiotemporal metabolomics (Control—LPS-8 h—LPS-24 h, N = 4). Moreover, a clinical cohort including 95 urinary samples (20 healthy *vs.* 30 sepsis *vs.* 45 SA-AKI) was recruited to validate the potential of 3-MH for SA-AKI diagnoses. Finally, we developed a model combined 3-MH and clinical variables via logistic regression (LR) to screen SA-AKI from patients with sepsis and assess the severity of disease.

### SA-AKI mice construction

Male C57BL/6 mice (aged 8–10 weeks, weighing 25–30 g) were purchased from Charles River Laboratory Animal Technology Co. Ltd. (Beijing, China). They were housed under standard conditions, with free access to food and water, and maintained on a 12-h light/12-h dark cycle at 22 °C. All experiments were approved by the Ethics Committee of the First Affiliated Hospital of Harbin Medical University (Ethical Approval Number: 2023022) and strictly followed the guidelines outlined in the Guide for the Care and Use of Laboratory Animals.

Mice were assigned to two parts. In the first part, twelve mice were divided into 3 groups, with 4 mice in each group. They were sacrificed at 8 and 24 h following the injection of LPS (10 mg/kg, *i.p.*). The control group, serving as a control, received an intraperitoneal injection of equal volume of 0.9% saline. In the second part, six mice received LPS (10 mg/kg, *i.p.*) and were euthanized at 24 h, and the other six mice received an intraperitoneal injection of equal volume of 0.9% saline. The detailed construction process of SA-AKI mice and real-time glomerular filtration rate (RT-GFR) measurement were described in the Supplementary material.

### Patient recruitment and sample collection

This study utilized a retrospective cohort design. Patients admitted to the intensive care unit (ICU) of the First Affiliated Hospital of Harbin Medical University between June 2023 and December 2024 were enrolled who met the Sepsis-3 criteria [1] for sepsis (n = 30) or the Kidney Disease Improving Global Outcomes (KDIGO) criteria [6, 7] for SA-AKI (n = 45). Healthy volunteers (n = 20) were recruited from the hospital's physical examination center. The urinary samples were collected after the clinical diagnosis of SA-AKI or sepsis.

The inclusion criteria were patients attending ICU developing sepsis or SA-AKI during their stay. Furthermore, patients need to satisfy the following standards: (1) age ≥ 18. (2) Patients with sepsis meeting the Sepsis-3 criteria [1], which include suspected infection and organ dysfunction as indicated by Sequential Organ Failure Assessment (SOFA) score ≥ 2. (3) AKI was defined based on serum creatinine (SCr) criteria of the KDIGO guidelines [6, 7]. The urine output criteria were not adopted to identify AKI since we only measured the urine output every 12 h instead of the hourly urine output data. The baseline SCr was determined as the lowest available value within hospitalization. The guidelines include an increasing in SCr of 0.3 mg/dL (≥ 26.5 μmol/L) within 48 h, or an increasing to more than 1.5 times the baseline value.

The exclusion criteria were: (1) discharge or death within 48 h after ICU admission. (2) chronic kidney disease (CKD) or other known kidney disease. (3) receive renal replacement therapy before admission (*e.g.*, hemodialysis, peritoneal dialysis). (4) kidney transplantation or nephrectomy. (5) serious disease, such as malignant tumor, autoimmune disease, acquired immune deficiency disease. (6) pregnant or lactating women and (7) insufficient clinical information.

The study was approved by the Ethics Committee of the First Affiliated Hospital of Harbin Medical University (Ethical Approval Number: IRB-AF/SC-04/02.0), and strictly conducted in accordance with the ethical principles ruled by the Declaration of Helsinki.

### Omics research

The detailed metabolomics sequencing method and data quality control methods were described in the Supplementary material [28–41].

### Statistical analysis

Continuous variables met normal distribution were expressed as Mean ± standard deviation (SD), student’s t-test (two tailed) was performed to compare differences between two groups and One-way ANOVA followed by Tukey test was adopted for multiple group comparisons. For non-normally distributed variables, data were presented as median (P25, P75), Mann–Whitney U tests were used to compare differences between two groups, while Kruskal–Wallis rank-sum test was employed for multiple group comparisons. Categorical variables were presented as frequencies or percentages, and Chi-Square Tests were employed to evaluate the differences between groups.

Renal and urinary metabolomics were analyzed via student’s t-test (two tailed) and Orthogonal Partial Least Squares—Discriminant Analysis (OPLS-DA). The significant threshold of differential analysis was set to *p* < 0.05, FC > 1.5 or FC < 1/1.5 and Variable Importance in the Projection (VIP) > 1. Kyoto Encyclopedia of Genes and Genomes (KEGG) enrichment analysis serves as a valuable tool for investigating metabolites functions.

Spearman correlation analysis was used to assess the associations between 3-MH and clinical indicators, with |R|> 0.5 and *p* < 0.05 indicating strong correlations. Univariate and multivariate regression were applied to evaluate the impact of 3-MH or clinical variables on SA-AKI, and odds ratio (OR) was applied as a quantitative index to describe the effects.

The receiver operating characteristic (ROC) curves of 3-MH was depicted and area under the receiver operating characteristic curve (AUC-ROC) were calculated. Clinical cohort were randomly divided into training (70%) and test (30%) sets. Logistic Regression (LR) was applied to train diagnostic model via threefold cross validation. Model performance was robustly estimated via AUC-ROC, area under the precision-recall curve (PR-AUC), sensitivity, specificity, recall, precision and F1 score through tidymodels package (v1.2.0) [42]. Statistical analysis and modeling procedures were performed via RStudio (v4.2.2).

## Results

### SA-AKI mice establishment and renal temporal metabolomics analysis

**Fig. 2:**
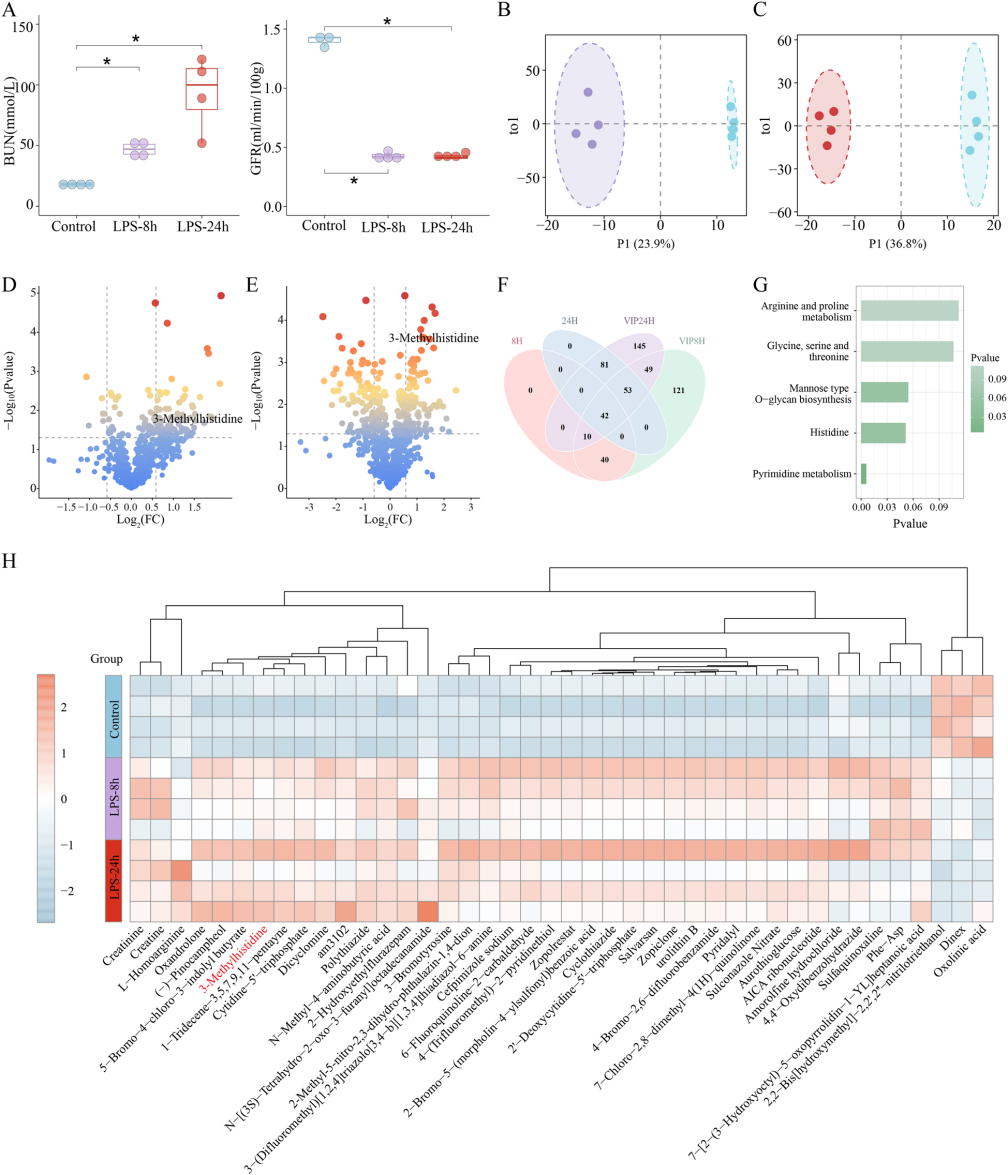
Systematic Analysis of Metabolic Activity Differences Between Renal Time Series.** A** Box plots present the biochemical indicators in control and LPS induced SA-AKI mice including BUN and GFR (Control—LPS-8 h—LPS-24 h, N = 4). **B, C** OPLS-DA score plot in control group and LPS-8 h group or LPS-24 h group. **D, E** Differential analysis of renal metabolomics between control group and LPS-8 h group or LPS-24 h group. Colors of points correspond to *p*-values. Significant after two-tailed t-test (*p* < 0.05) and FC > 1.5 & FC < 1/1.5. **F** Forty-two key renal metabolites were observed via intersecting differential analysis and OPLS-DA analysis. **G, H** The KEGG enrichment and Heatmap of key renal metabolites. *, **, and *** indicate *p* < 0.05, *p* < 0.01, and *p* < 0.001, respectively.

In the first part, blood urea nitrogen (BUN) and glomerular filtration rate (GFR) changed significantly from 0 to 8 h to 24 h (*p* < 0.05, ANOVA test). Specifically, BUN increased from 0 to 8 h and from 0 to 24 h (*p* < 0.05) while GFR decreased from 0 to 8 h and 0 h to 24 h (*p* < 0.05) (Fig. 2A). In the second part, compared with control group, urinary KIM-1 levels were significantly elevated in the SA-AKI group (*p* < 0.05) (Figure S1). Combined with BUN, GFR and urinary KIM-1, which indicated that the SA-AKI mice were successfully established.

The metabolic activity significantly changed from control to LPS-8 h to LPS-24 h (Fig. 2B, C). Compared with control group, 92 differential metabolites were identified in the LPS-8 h group, including 86 up-regulated metabolites and 6 down-regulated metabolites (FC > 1.5 & FC < 1/1.5, *p* < 0.05) (Fig. 2D; Table S1). These differential metabolites were primarily enriched in energy metabolism pathways, particularly the citrate cycle (TCA cycle) (Figure S2). Previous studies have shown that renal tubular epithelial cells (RTECs) under acute kidney injury exhibit increased aerobic glycolysis and reduced mitochondrial oxidative phosphorylation (OXPHOS), leading to excessive reactive oxygen species (ROS) accumulation, which could exacerbate energy depletion and promote cell apoptosis [43–45]. Targeting the TCA cycle pathway could restore energy homeostasis, providing a potential therapeutic strategy for SA-AKI.

Similarly, compared to control group, 176 differential metabolites were identified in the LPS-24 h group, which included 87 up-regulated metabolites and 89 down-regulated metabolites (FC > 1.5 & FC < 1/1.5, *p* < 0.05) (Fig. 2E). The differential renal metabolites were predominantly involved in nucleotide metabolism such as purine metabolism and pyrimidine metabolism (Figure S3). Uric acid, the terminal product of purine metabolism [46], has been demonstrated to activate the NOD-like receptor family, pyrin domain-containing 3 (NLRP3) inflammasome and exacerbate inflammatory responses [47, 48].

Forty-two key renal metabolites were obtained from the intersection of differential analyses and Orthogonal Partial Least Squares—Discriminant Analysis (OPLS-DA) (Fig. 2F, H). These metabolites concentrated in amino acid metabolism pathways such as arginine and proline metabolism, glycine, serine, and threonine metabolism, as well as histidine metabolism (Fig. 2G), which might contribute to SA-AKI progression through multiple mechanisms. For instance, arginine deficiency could lead to impaired T cell function [49], while arginine supplementation could improve renal perfusion [50].

### Renal and urinary metabolomics analysis identifies 3-methylhistidine (3-MH)

**Fig. 3:**
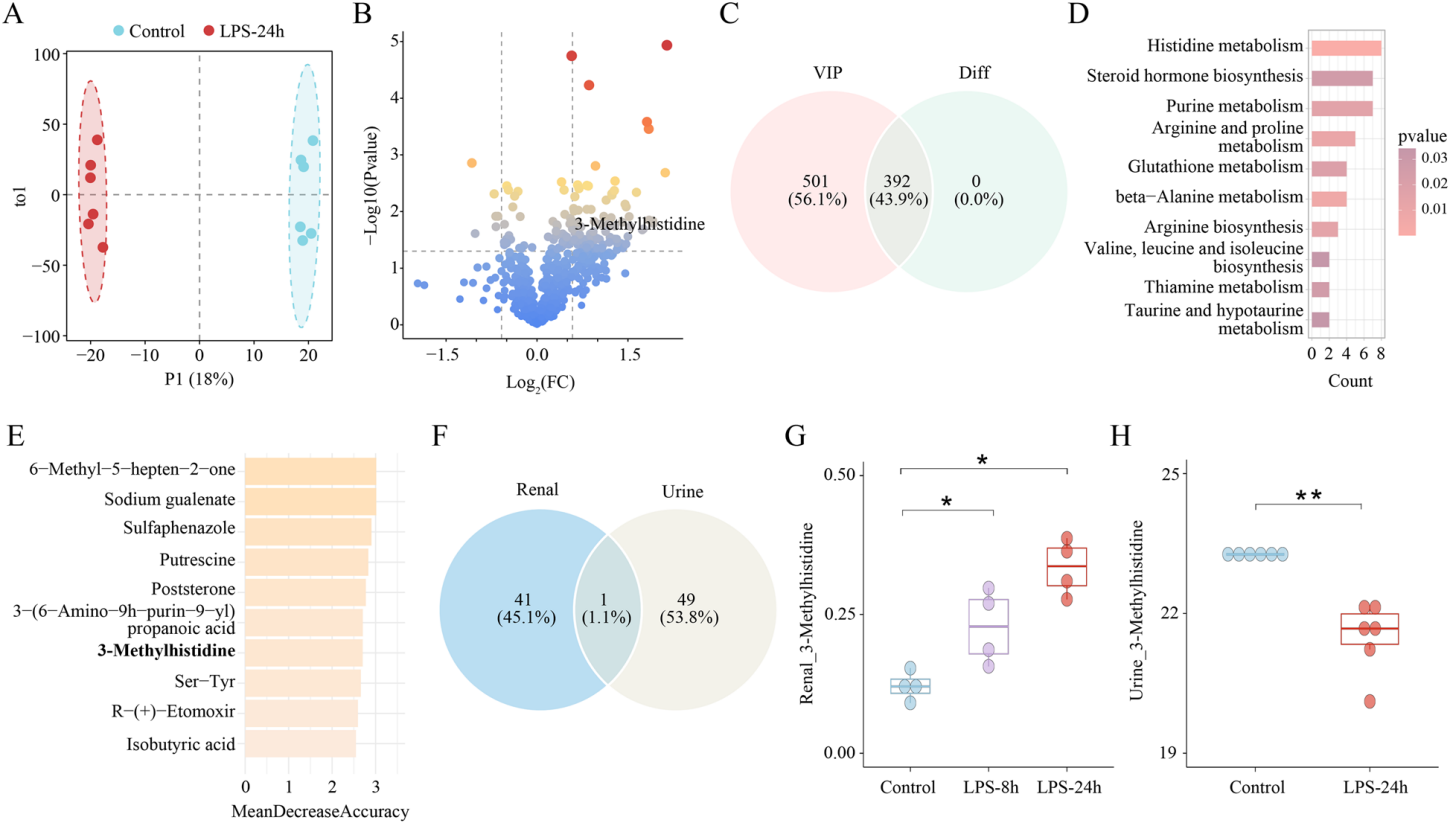
Integrated Analysis of Mice Urinary Metabolomics and Renal Temporal Metabolomics. **A** OPLS-DA score plot between the control group and LPS-24 h group (N = 6). **B** Differential analysis of renal metabolomics between control group and LPS-24 h group. Colors of points correspond to *p*-values. Significant after two-tailed t-test (*p* < 0.05) and FC > 1.5 & FC < 1/1.5. **C** Differential urinary metabolites were observed via intersecting differential analysis and OPLS-DA analysis. **D** The KEGG enrichment of differential urinary metabolites. **E** The top 10 urinary metabolites were calculated based on Mean Decrease in Accuracy via the Random Forest algorithm. **F** 3-Methylhistidine (3-MH) was identified via key renal metabolites and top 50 urinary metabolites. **G** The time variation of renal 3-MH in SA-AKI mice at different time points (Control, LPS-8 h, LPS-24 h). **H** Box plot presents urinary 3-MH in control group and LPS-24 h group. *, **, and *** indicate *p* < 0.05, *p* < 0.01, and *p* < 0.001, respectively.

There was an obvious change in urinary metabolic activity between LPS-24 h mice and control mice (Fig. 3A). Compared to control group, 392 metabolites were significantly changed in LPS-24 h group, including 90 up-regulated metabolites and 302 down-regulated metabolites (*p* < 0.05, FC > 1.5 & FC < 1/1.5) (Fig. 3B; Table S2). Based on OPLS-DA analysis and differential analysis, 392 urinary metabolites were detected (Fig. 3C).

Consistent with the renal metabolic profile, the differentially urinary metabolites were similarly enriched in amino acid metabolism pathways (Fig. 3D). For example, 3-methylhistidine (3-MH), a marker of histidine metabolism that reflects muscle protein breakdown [51]. Increased serum 3-MH levels suggest enhanced muscle catabolism. The accumulation of free amino acids could dysregulate immune responses through altered nutrient signaling pathway (e.g., mTOR or AMPK pathways) [52]. These interconnected metabolic pathways might collectively exacerbate the progression of SA-AKI through impairing renal energy homeostasis and amplifying inflammatory injury.

The top 50 metabolites were determined based on Mean Decrease Accuracy (MDA) algorithm (Table 1, Fig. 3E). Eventually, 3-MH was identified as a potential biomarker via intersecting key renal metabolites and the top 50 urinary metabolites (Fig. 3F). 3-MH exhibited an increasing in renal temporal metabolomics in the LPS-8 h group (FC = 1.88, *p* < 0.05) and LPS-24 h group (FC = 2.76, *p* < 0.05) compared to the control group (Fig. 3G). Renal 3-MH was negatively with GFR (R = − 0.65, *p* < 0.05) (Figure S4) while positively with BUN (R = 0.85, *p* < 0.001) (Figure S5). Conversely, urinary 3-MH levels significantly decreased in the LPS-24 h group (FC = 0.16, *p* < 0.05) (Fig. 3H).

**Table 1:** The top 50 urinary metabolites

Name	CAS-ID	Log_2_(FC)	VIP	MDA
6-Methyl-5-hepten-2-one	110-93-0	− 1.04	2.08	3.01
Sodium gualenate	6223-35-4	− 2.93	2.03	3.01
Sulfaphenazole	526-08-9	− 1.92	1.98	2.90
Putrescine	110-60-1	− 0.99	1.76	2.83
Poststerone	10162-99-9	− 1.31	1.92	2.78
3-(6-Amino-9 h-purin-9-yl)propanoic acid	4244-47-7	− 4.13	2.13	2.71
3-Methylhistidine	368-16-1	− 1.77	2.07	2.70
Ser-Tyr	21435-27-8	− 2.23	1.82	2.66
R-(+)-Etomoxir	124083-20-1	7.11	2.03	2.59
Isobutyric acid	79-31-2	− 0.71	2.08	2.55
N,N-Dimethylarginine	30315-93-6	− 1.44	1.98	2.54
Asn-Arg	2478/1/5	− 1.06	2.14	2.54
Imidazole	288-32-4	0.69	1.97	2.49
CYCLOPIAZONIC ACID	18172-33-3	− 1.01	2.02	2.49
3,4,5-Trimethoxycinnamic Acid	90-50-6	− 1.21	1.94	2.48
Tilarginine Acetate	53308-83-1 | 17035-90-4	1.58	2.07	2.42
Thiamine	59-43-8	− 1.62	2.11	2.41
Benfotiamine	22457-89-2	− 1.61	1.81	2.41
2,6-Dimethylphenol	576-26-1	− 0.86	1.80	2.38
3-(2-Hydroxyphenyl)propanoic acid	495-78-3	4.01	2.09	2.20
Biopterin	22150-76-1	1.44	1.77	2.17
Mesaconic acid	498-24-8	− 1.46	2.09	2.17
Pro-Ser	71835-80-8	− 1.44	1.98	2.17
4-Methoxysalicylic Acid	2237-36-7	− 1.22	1.93	2.16
Cosmosiin	578-74-5	3.02	1.88	2.16
(4-Hydroxy-3-methoxyphenyl)ethanol	2380-78-1	− 1.37	1.90	2.12
Cryptotanshinone	35825-57-1 | 4733-35-1	− 1.14	1.93	2.00
(S)-Verimol F	212516-39-7	− 1.61	1.73	1.99
Epinephrine	51-43-4	− 1.40	1.99	1.99
Ramipril	87333-19-5	− 2.26	1.84	1.99
4-O-beta-D-glucosyl-4-coumaric acid	14364-05-7	− 2.54	1.73	1.98
2'-Deoxyinosine	890-38-0	2.92	2.04	1.97
Probenazole	27605-76-1	− 1.53	2.00	1.97
2-Aminoethanesulfinic Acid	300-84-5	− 1.83	2.05	1.97
Geranyl acetate	105-87-3	− 1.49	1.79	1.94
Imiquimod	99011-02-6	− 1.09	1.37	1.92
Xylose	58-86-6	3.30	2.20	1.91
dl-Arterenol hydrochloride	55-27-6 | 329-56-6	− 0.64	1.95	1.85
Corticosterone	50-22-6	1.91	2.11	1.73
Trigonelline	535-83-1	− 1.03	1.90	1.73
DL-3-Phenyllactic acid	828-01-3	− 1.29	1.87	1.73
3-Methylxanthine	1076-22-8	− 0.80	1.68	1.73
1-methylhistamine	501-75-7	1.13	1.96	1.73
Hildecarpin	99624-64-3	− 0.98	1.77	1.72
11-Dehydro-2,3-dinor-txb2	79250-60-5	− 1.76	1.98	1.72
beta-D-Galp-(1- > 3)-D-GalpNAc	20972-29-6	− 2.68	1.79	1.71
DL-3,4-Dihydroxyphenyl glycol	28822-73-3	− 0.62	1.55	1.71
2,3-Dimethoxyphenol	5150-42-5	− 0.80	1.86	1.71
2-Chloro-1,4-naphthoquinone	1010-60-2 | 96843-34-4	1.52	1.89	1.70
(2-Hydroxyethyl)phosphonic acid	22987-21-9	− 1.05	1.73	1.70

CAS Chemical Abstracts Service, FC Fold Change, VIP Variable Importance in the Projection, MDA MeanDecreaseAccuracy

### Renal spatiotemporal metabolomics investigation of 3-MH in SA-AKI mice

**Fig. 4:**
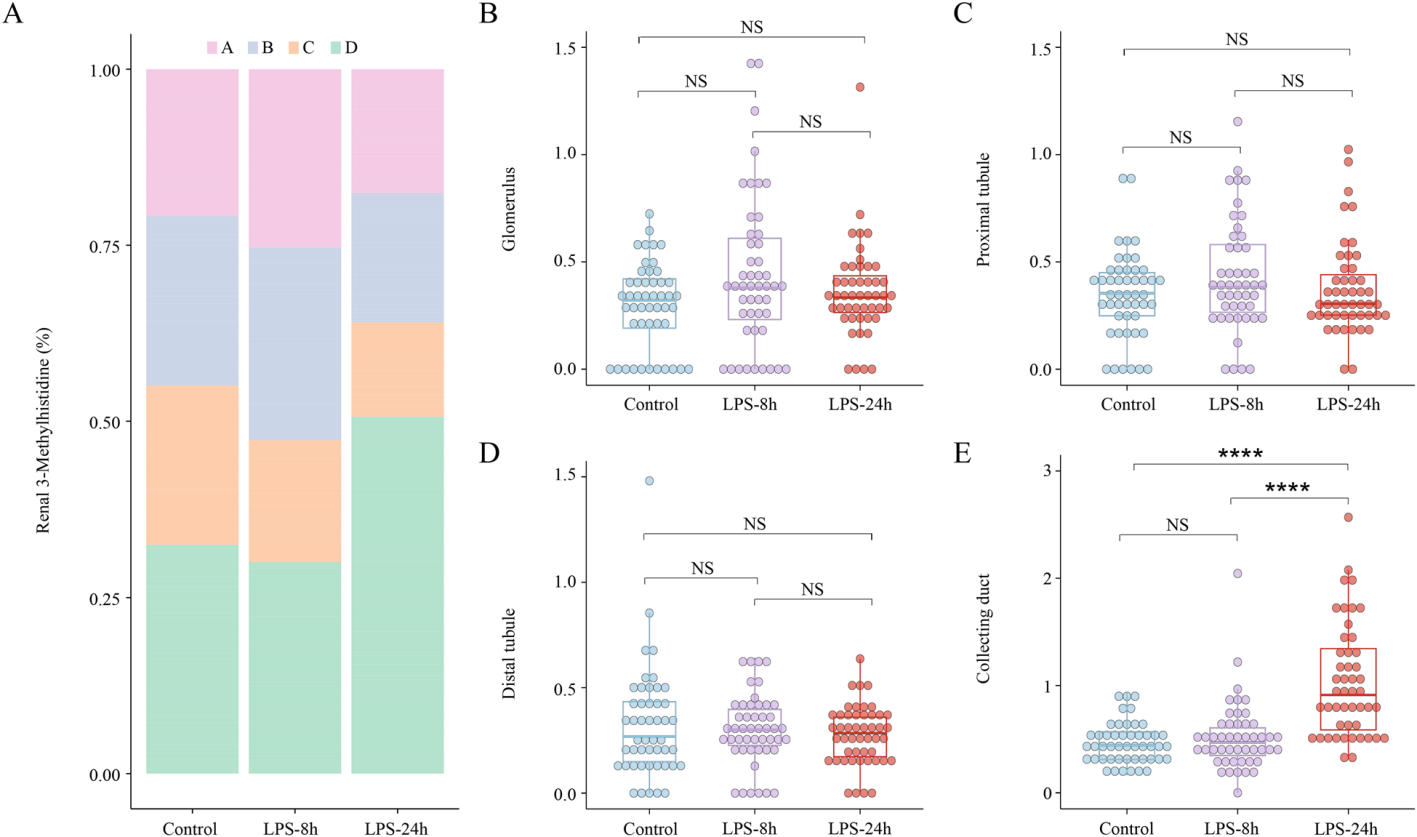
Renal Spatiotemporal Dynamics of 3-Methylhistidine. **A** The percentage of 3-Methylhistidine (3-MH) in different renal regions including glomeruli, proximal tubules, distal tubules and collecting ducts in SA-AKI mice (Control—LPS-8 h—LPS-24 h, N = 4). **B–E** Metabolic activity of 3-MH at each renal region in time series. **** represents for *p* < 0.0001.

We performed renal spatiotemporal metabolomics analyses of SA-AKI mice at three time points (0 h, 8 h, 24 h) to investigate the specific spatial distribution of 3-MH. 3-MH displayed an average distribution across the glomerulus, proximal convoluted tubules, distal convoluted tubules, and collecting tubules in the control group, with no significant changes in the LPS-8 h group. However, 3-MH showed major distribution at the collecting ducts in the LPS-24 h group (Fig. 4A). At the same time, the metabolic activity of 3-MH in glomerulus, proximal convoluted tubules, and distal convoluted tubules remained stable (Fig. 4B–D). Notably, 3-MH exhibited a distinct trend in the LPS-8 h (FC = 1.98, *p* < 0.0001) and LPS-24 h (FC = 2.22, *p* < 0.0001) (Fig. 4E; Table S3).

### Quantitative urinary metabolomics validation 3-MH as a potential biomarker

A clinical cohort involved 95 individuals (20 healthy *vs.* 30 sepsis *vs.* 45 SA-AKI) was recruited to validate urinary 3-MH as a diagnostic biomarker for SA-AKI. Among patients with SA-AKI, there were 13 patients (28.9%) in stage I, 16 patients (35.6%) in stage II, and 16 patients (35.6%) in stage III (Table 2). The major infection sites in patients with SA-AKI were the lungs (n = 10, 22.2%), peritoneum (n = 9, 20.0%), biliary tract (n = 6, 13.3%), intestinal (n = 6, 13.3%) and liver (n = 6, 13.3%) while in patients with sepsis were lungs (n = 12, 40.0%), peritoneum (n = 6, 20.0%) and biliary tract (n = 4, 13.3%) (Table 2). As for the main diagnoses, severe pneumonia was the most prevalent condition in patients with SA-AKI (n = 8, 17.8%) or sepsis (n = 8, 26.7%). Gastrointestinal perforation was another notable primary disease, accounting for 8.9% (n = 4) in SA-AKI group and 16.7% (n = 5) in sepsis group. Notably, Liver abscess was more prevalent in the SA-AKI group (13.3%, n = 6) than in the sepsis group (3.3%, n = 1) (Table 2). Furthermore, compared with patients with sepsis, patients with SA-AKI exhibited more severe clinical conditions and organ dysfunction, as evidenced by significantly higher the Acute Physiology and Chronic Health Evaluation II (APACHE II) scores (23 ± 5 *vs.* 19 ± 7, *p* = 0.011), Sequential Organ Failure Assessment (SOFA) scores (10.0 [9.0–13.0] *vs.* 8.0 [7.0–10.0], *p* = 0.008), and significantly elevated procalcitonin (PCT) levels (36 [20–95] *vs.* 16 [1–31] ng/mL,* p* < 0.001) (Table 2). In addition, the platelet (PLT) count (88 [56, 168] *vs.* 210 [149, 283] 10^9^/L, *p* < 0.001) and lymphocyte (LYMPH) count (0.71 [0.52, 0.90] *vs.* 1.01 [0.73, 1.49] 10^9^/L, *p* = 0.002) in patients with SA-AKI was significantly decreased (Table 2).

**Table 2:** Baseline characteristics of clinical cohort

	Health (N = 20)	Sepsis (N = 30)	SA-AKI (N = 45)	P-value
Age	59 (47,64)	50 (43,63)	61 (51,68)	0.150^2^
Sex				0.973^3^
Male	12 (60%)	17 (56.7%)	26 (57.8%)	
Female	8 (40%)	13 (43.3%)	19 (42.2%)	
AKI stages				-
I	–	–	13 (28.9%)	
II	–	–	16 (35.6%)	
III	–	–	16 (35.6%)	
APACHEII, Mean ± SD, [IQR]	–	19 ± 7	23 ± 5	0.011^5^
SOFA, median, [IQR]	–	8.0 (7.0, 10.0)	10.0 (9.0, 13.0)	0.008^4^
PCT (ng/mL), median, [IQR]	–	16 (1, 31)	36 (20, 95)	< 0.001^4^
WBC (10^9^/L), median, [IQR]	–	12 (7, 16)	11 (7, 14)	0.654^4^
Neutrophil (10^9^/L), median, [IQR]	–	10 (6, 15)	9 (6, 14)	0.499^4^
LYMPH# (10^9^/L), median, [IQR]	–	1.01 (0.73, 1.49)	0.71 (0.52, 0.90)	0.002^4^
Platelet (10^9^/L), median, [IQR]	–	210 (149, 283)	88 (56, 168)	< 0.001^4^
PH, median, [IQR]	–	7.41 (7.34, 7.45)	7.42 (7.32, 7.44)	0.729^4^
PaCO_2_, (mmHg), median, [IQR]	–	34 (28, 41)	31 (27, 37)	0.263^4^
PaO_2_, (mmHg), median, [IQR]	–	90 (74, 130)	94 (75, 129)	0.901^4^
Lac (mmol/L), median, [IQR]	–	3.25 (1.60, 4.75)	2.20 (1.40, 3.90)	0.468^4^
HCO3^−^ (mmol/L), median, [IQR]	–	21.4 (18.8, 23.7)	19.7 (17.0, 22.8)	0.245^4^
ALT (U/L), median, [IQR]	–	20 (12, 40)	34 (19, 199)	0.015^4^
AST (U/L), median, [IQR]	–	32 (22, 67)	66 (32, 206)	0.016^4^
TBIL (µmol/L), median, [IQR]	–	17 (9, 31)	24 (14, 89)	0.112^4^
DBIL (µmol/L), median, [IQR]	–	7 (4, 15)	12 (8, 55)	0.019^4^
SCr (µmol/L L), median, [IQR]	–	78 (68, 104)	214 (154, 330)	< 0.001^4^
Urea (mg/dL), median, [IQR]	–	6 (5, 10)	15 (12, 23)	< 0.001^4^
Mechanical Ventilation	–			0.147^3^
Yes		24 (80.0%)	29 (64.4%)	
No		6 (20.0%)	16 (35.6%)	
28-day death	–			0.694^3^
Yes		10 (33.3%)	17 (37.8%)	
No		20 (66.7%)	28 (62.2%)	
Infectious site				0.764^6^
Biliary tract	–	4 (13.3%)	6 (13.3%)	
Chest Wall	–	0 (0.0%)	1 (2.2%)	
Intestinal	–	1 (3.3%)	6 (13.3%)	
Liver	–	2 (6.7%)	6 (13.3%)	
Lungs	–	12 (40.0%)	10 (22.2%)	
Pancreas	–	2 (6.7%)	3 (6.7%)	
Pericardium	–	0 (0.0%)	1 (2.2%)	
Peritoneum	–	6 (20.0%)	9 (20.0%)	
Skin	–	1 (3.3%)	1 (2.2%)	
Urinary tract	–	2 (6.7%)	2 (4.4%)	
Primary diagnosis				-
Acute cholangitis	–	1 (3.3%)	4 (8.9%)	
Acute pericarditis	–	0 (0.0%)	1 (2.2%)	
Acute peritonitis	–	1 (3.3%)	3 (6.7%)	
Cellulitis	–	0 (0.0%)	1 (2.2%)	
Cerebral hemorrhage	–	1 (3.3%)	0 (0.0%)	
Cerebral hernia	–	1 (3.3%)	0 (0.0%)	
Cerebral infarction	–	2 (6.7%)	0 (0.0%)	
Chest Wall abscess	–	0 (0.0%	1 (2.2%)	
Cholecystitis	–	3 (10.0%)	2 (4.4%)	
Gastrointestinal bleeding	–	0 (0.0%)	1 (2.2%)	
Gastrointestinal perforation	–	5 (16.7%)	4 (8.9%)	
Hepatic encephalopathy	–	1 (3.3%)	0 (0.0%)	
Intestinal diverticulum	–	0 (0.0%)	1 (2.2%)	
Intestinal necrosis	–	0 (0.0%)	3 (6.7%)	
Intestinal obstruction	–	1 (3.3%)	2 (4.4%)	
Liver abscess	–	1 (3.3%)	6 (13.3%)	
Maxillofacial space infections	–	1 (3.3%)	0 (0.0%)	
Primary peritonitis	–	0 (0.0%)	2 (4.4%)	
Pulmonary contusion	–	0 (0.0%)	1 (2.2%)	
Renal abscess	–	2 (6.7%)	1 (2.2%)	
Severe pancreatitis	–	2 (6.7%)	3 (6.7%)	
Severe pneumonia	–	8 (26.7%)	8 (17.8%)	
Ureteral calculus	–	0 (0.0%)	1 (2.2%)	

^1^Median (IQR); n (%); Mean ± SD, ^2^Kruskal-Wallis rank sum test, ^3^Pearson's Chi-squared test, ^4^Wilcoxon rank sum test, ^5^Welch Two Sample t-test, ^6^ Fisher’s exact test

APACHE II Acute Physiological and Chronic Health Assessment, SOFA Sequential Organ Failure Assessment, PCT Procalcitonin, WBC white blood cell, LYMPH# Lymphocytes; Lac Lactic acid, ALT Alanine aminotransferase, AST Aspartate aminotransferase, TBIL Total bilirubin, DBIL Direct bilirubin, SCr Serum creatinine

**Fig. 5:**
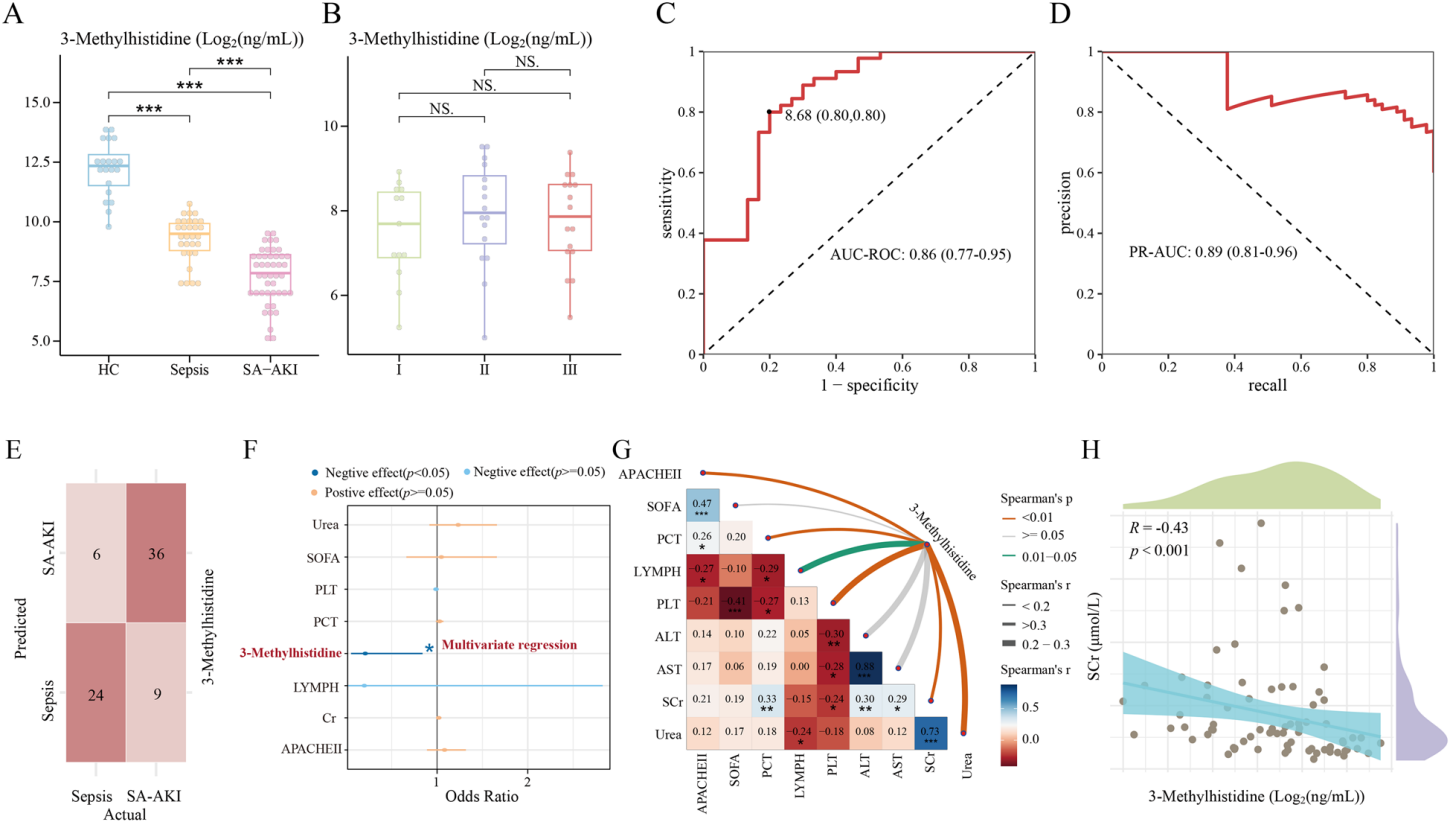
Employed quantitative metabolomics sequencing to validate urinary 3-Methylhistidine (3-MH) as a potential diagnostic biomarker for SA-AKI. **A, B** Abundance distribution of urinary 3-MH in clinical cohort (Health volunteers *vs.* sepsis patients *vs.* SA-AKI patients, N = 95) or in patients with different stages of SA-AKI (13 Stage I *vs.* 16 Stage II *vs.* 16 Stage III, N = 45). **C, D** The Receiver Operating Characteristic (ROC) curve **(C)** and Precision-Recall (PR) curve **(D)** demonstrated the diagnostic ability of urinary 3-MH for SA-AKI. **E** Confusion matrix of 3-MH. **F** Multivariate regression analysis in 3-MH and clinical variables. **G** Heatmap illustrated the relationships among clinical factors and the correlations between 3-MH and each clinical factor. The color and thickness of lines connecting 3-MH represent spearman significance (brown, green and gray indicates *p* < 0.01, 0.01 ≤ *p* < 0.05, *p* ≥ 0.05) and the absolute value of spearman coefficient, the color intensity of heatmap represents the absolute value of spearman coefficient among clinical factors. **H** Correlation analysis between 3-MH and serum creatinine (SCr). *, **, and *** indicate *p* < 0.05, *p* < 0.01, and *p* < 0.001, respectively.

Urinary 3-MH decreased gradually during the progression of health—sepsis—SA-AKI (*p* < 0.05, ANOVA) (Fig. 5A; Table S4). Specifically, patients with sepsis exhibited significantly lower 3-MH levels compared to the healthy volunteers (9.27 ± 0.94 *vs.* 12.21 ± 1.15 log_2_(ng/mL), N = 50, *p* < 0.001). Moreover, the levels of 3-MH in patients with SA-AKI were lower than those with sepsis (7.74 ± 1.15 *vs.* 9.27 ± 0.94 log_2_(ng/mL), N = 75, *p* < 0.001) and the healthy volunteers (7.74 ± 1.15 *vs.* 12.21 ± 1.15 log_2_(ng/mL), N = 65, *p* < 0.001) (Fig. 5A). However, there was no change in the stage I-II-III in the patients with SA-AKI (*p* > 0.05, ANOVA) (Fig. 5B).

Urinary 3-MH demonstrated strong diagnostic performance for distinguishing SA-AKI from patients with sepsis, with an area under the receiver operating characteristic curve (AUC-ROC) of 0.86 (95% CI 0.77–0.95) and an area under the precision-recall curve (PR-AUC) of 0.89 (95%CI 0.81–0.96) (Fig. 5C, D). The optimal diagnostic cutoff for urinary 3-MH was identified as 8.68 log₂(ng/mL) based on Youden’s index, yielding a sensitivity of 0.80, specificity of 0.80, recall of 0.80, precision of 0.86 and an F1 score of 0.83 (Fig. 5C-E, Table 3).

**Table 3:** The diagnostic performance of urinary 3-Methylhistidine and logistic regression model

	3-Methylhistidine	Logistic regression model	
	–	Train dataset	Test dataset
ROC-AUC	0.86	0.88	0.89
PR-AUC	0.89	0.91	0.95
Accuracy	0.80	0.86	0.83
Sensitivity	0.80	0.84	0.79
Specificity	0.80	0.86	0.89
Recall	0.80	0.84	0.79
Precision	0.86	0.90	0.92
F1 Score	0.83	0.87	0.85

ROC-AUC Receiver Operating Characteristic—Area Under the Curve, PR-AUC Precision-Recall—Area Under the Curve

Urinary 3-MH (OR = 0.23, 95% CI: 0.10–0.42, *p* < 0.001), LYMPH count (OR = 0.49, 95% CI: 0.22–0.92, *p* < 0.05), and PLT count (OR = 0.99, 95% CI: 0.99–1.00, *p* < 0.01) were identified as factors which were negatively associated for SA-AKI occurrence (Figure S6). On the other hand, SCr level (OR = 1.03, 95% CI: 1.02–1.05, *p* < 0.001), PCT (OR = 1.04, 95% CI: 1.02–1.06, *p* < 0.001), APACHE II score (OR = 1.13, 95% CI: 1.04–1.25, p < 0.05), SOFA score (OR = 1.18, 95% CI: 1.02–1.39, *p* < 0.05), and blood urea (OR = 1.26, 95% CI: 1.13–1.44, *p* < 0.001) were recognized as factors which were positively associated with SA-AKI occurrence (Figure S6). Moreover, lower urinary 3-MH (OR = 0.21, 95% CI: 0.05–0.84, *p* < 0.05) was strongly correlated with SA-AKI after multivariate adjustment for APACHE II score (OR = 1.08, 95% CI: 0.89–1.32, *p* > 0.05), SCr level (OR = 1.02, 95% CI: 1.00–1.04, *p* > 0.05), LYMPH count (OR = 0.20, 95% CI: 0.01–2.83, *p* > 0.05), PCT (OR = 1.03, 95% CI:0.99–1.07, *p* > 0.05), PLT count (OR = 0.99, 95% CI: 0.98–1.00, *p* > 0.05), SOFA score (OR = 1.05, 95% CI: 0.66–1.66, *p* > 0.05), and blood urea (OR = 1.23, 95% CI: 0.92–1.66, *p* > 0.05) (Fig. 5F). Urinary 3-MH levels demonstrated significant correlations with multiple clinical indicators (Fig. 5G), for instance the contrary tendency was found between 3-MH and serum creatinine in our research (R = − 0.43, *p* < 0.001) (Fig. 5H).

### Combined 3-MH and clinical variables to construct a diagnostic model

**Fig. 6:**
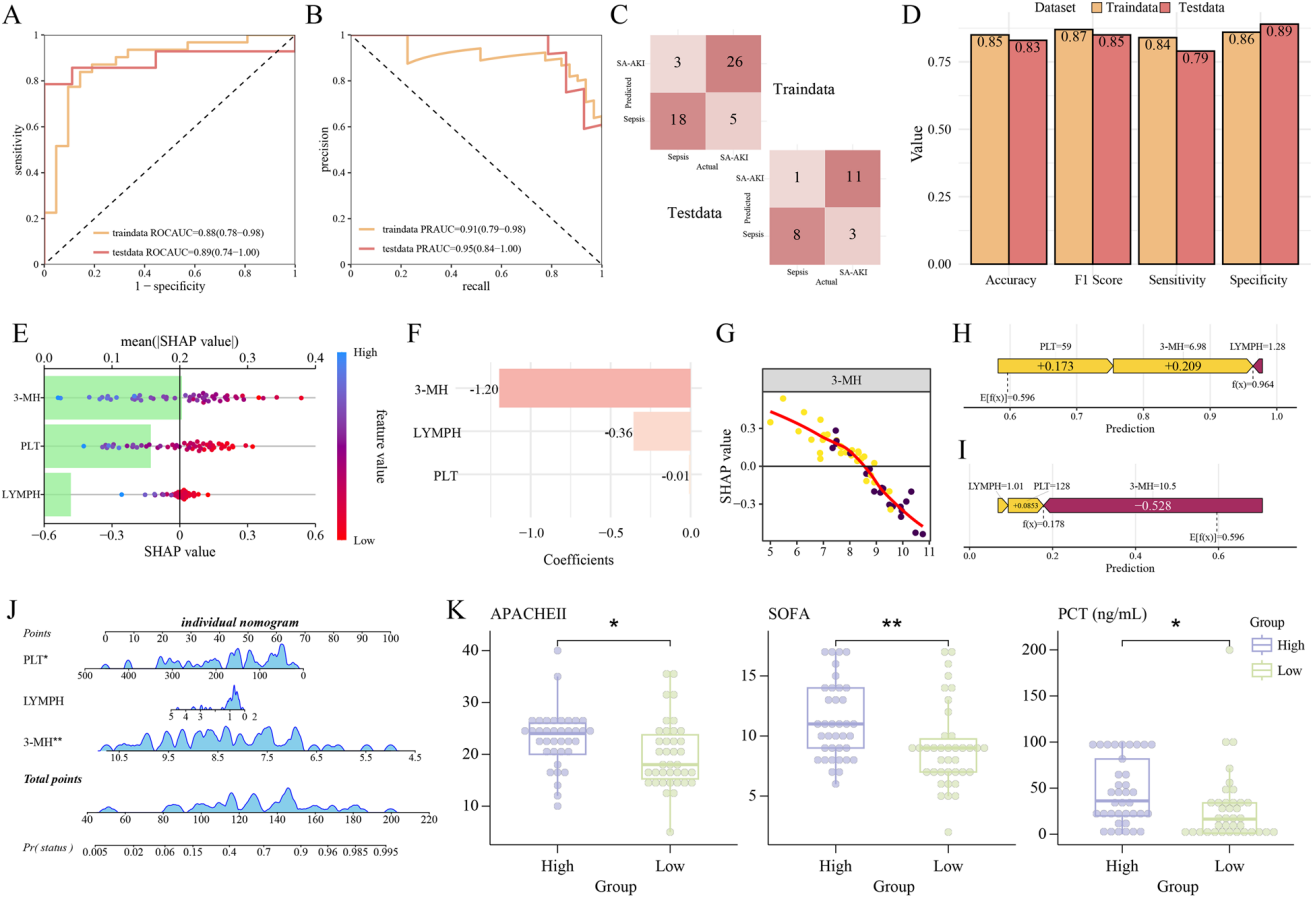
Construction of the diagnostic model combined 3-Methylhistidine (3-MH) with clinical variables.** A–C** The Receiver Operating Characteristic (ROC) curve **(A)**, Precision-Recall (PR) curve **(B)** and confusion matrix **(C)** in the train set and test set. **D)** Box plot illustrated the value of accuracy, F1 score, sensitivity and specificity in train set and test set. **E–F** Meanshap **(E)** and coefficients **(F)** of urinary 3-MH, platelet (PLT) count and lymphocyte (LYMPH) count in the diagnostic model. **G** SHAP Partial dependence plot for urinary 3-MH. **H, I** SHAP force plot for patient with SA-AKI **(H)** or sepsis **(I)**. **J** Diagnostic model nomogram. **K** Distributions of APACHEII score, SOFA score and procalcitonin for the high-risk (purple) and low-risk (green) patients, which were stratified by the median of the nomogram score. * and ** indicate *p* < 0.05 and *p* < 0.01

A diagnostic model for SA-AKI was constructed incorporating urinary 3-MH, LYMPH count and PLT count via logistic regression (LR) algorithm. The diagnostic model demonstrated excellent discriminative capacity, evidenced by an AUC-ROC of 0.89 (95% CI: 0.74–1.00) (Fig. 6A) and a PR-AUC of 0.94 (95% CI: 0.84–1.00) (Fig. 6B). In addition, the model maintained robust performance with sensitivity (0.79) and specificity (0.89), accuracy (0.83) and F1 score (0.85) (Fig. 6C, D, Table 3). The model coefficients of 3-MH, LYMPH count and PLT were − 1.20, − 0.36 and − 0.01 (Fig. 6F).

Urinary 3-MH was identified as the most influential predictor (|mean SHAP|= 0.20) (Fig. 6E). Non-linear relationships were observed for 3-MH, PLT, and LYMPH with SA-AKI (Fig. 6G; Figure S7). Individual decision plot analysis demonstrated the discriminative capacity: for a representative SA-AKI patient (3-MH = 6.98 log_2_(ng/mL), PLT count = 59 (10^9^/L), LYMPH count = 1.28 (10^9^/L)), the predicted probability reached 0.964 (Fig. 6H), while for a sepsis patient (3-MH = 10.5 log_2_(ng/mL), PLT count = 128 (10^9/L), LYMPH count = 1.01 (10^9^/L)) it was 0.178, with an overall average predictive value of 0.596 (Fig. 6I).

Moreover, a nomogram was created to access each patient (Fig. 6J). Patients with sepsis or SA-AKI were categorized into high or low score groups based on the median score. APACHE II score, SOFA score and PCT were significantly higher in the high-score group compared to the low-score group (APACHE II score: 23 ± 6 *vs.* 20 ± 7, *p* < 0.05; SOFA: 11 [9, 14] *vs.* 9 [7, 10], *p* < 0.01; PCT: 36 [20,82] *vs.* 16 [1, 34] ng/mL, *p* < 0.01) (Fig. 6K), illustrating the diagnostic model is also an indicator for disease severity.

## Discussion

Through integrating renal temporal metabolomics and urinary metabolomics in lipopolysaccharide (LPS)-induced sepsis-associated acute kidney injury (SA-AKI) mice, 3-Methylhistidine (3-MH) was identified as a potential urinary biomarker for SA-AKI. Subsequently, we performed a renal spatiotemporal metabolomics to evaluate the specific distribution in collecting ducts of 3-MH. Moreover, a clinical cohort (20 healthy volunteers *vs.* 30 sepsis patients *vs.* 45 SA-AKI patients) was recruited to validate urinary 3-MH as a diagnostic molecule (AUC = 0.86, 95% CI: 0.77–0.95). Eventually, the nomogram score which 3-MH contributed most have the capacity to assess severity of disease.

3-MH derives from the catabolism of muscle proteins and exists predominantly as a free amino acid in circulation [51, 53]. Additionally, 3-MH is filtered by the kidneys and excreted into urine [54]. These distinct characteristics promote urinary 3-MH to serve as a potential biomarker for renal functional injury. Contrary to the previous research [55], Tiao et al. found that urinary 3-MH/creatinine was increased in post-surgery patients with sepsis compared to those without sepsis. However, it did not estimate how urinary 3-MH levels varied in septic patients. Additionally, urinary creatinine had been proved decreased in septic patients resulting from the reduction of tubular secretory function [56]. Therefore, the result might exhibit a tendency to reflect the decline of creatinine clearance rate instead of an increase in 3-MH secretion. Currently, the value of urinary 3-MH has not been explored in patients with SA-AKI. Our study revealed that renal 3-MH level exhibited a correlation with glomerular filtration rate (GFR) and blood urea nitrogen (BUN) in mice. Clinically, urinary 3-MH level demonstrated a gradual decline from healthy condition to sepsis and further to SA-AKI. These consistent results suggested that urinary 3-MH levels have the potential to serve as a biomarker for monitoring renal filtration function.

Notably, 3-MH was predominantly localized to the collecting ducts through renal spatiotemporal metabolomics sequencing, indicating the alteration of reabsorption function could be a potential determinant of its distribution. Currently, the definition of AKI stages is based on serum creatinine (SCr) or urine output, which primarily reflects glomerular filtration but fails to present the complex pathological process of SA-AKI [57–59]. This discrepancy in assessment focus has resulted in an inconsistency outcome between the levels of urinary 3-MH and SA-AKI stages defined by KDIGO guidelines [6, 7]. Specifically, the lack of significant differences in urinary 3-MH levels across different AKI stages might initially lead to the misconception that urinary 3-MH could not reflect renal injury. In fact, 3-MH was identified as a renal functional biomarker for SA-AKI, which could simultaneously reflect the changes in renal reabsorption function and filtration function. Moreover, cystatin C (Cys C) protein, a recognized renal functional biomarker [60], is freely filtered by glomeruli but entirely reabsorbed and degraded in renal tubules [60–62]. Serum Cys C protein could not evaluate the variation in renal reabsorption function. However, urinary 3-MH integrates both renal filtration and reabsorption functions, providing a more comprehensive assessment of renal functional status. Therefore, urinary 3-MH could be recognized as a superior functional indicator for SA-AKI.

Urinary 3-MH could be utilized as a diagnostic biomarker for SA-AKI in clinics. Reduced 3-MH level was relevant with higher SA-AKI risk, enabling it to serve as a diagnostic signature to guide clinical judgment. Although the urinary 3-MH level could not be directly applied for AKI staging or severity assessing, it can more comprehensively reflect the condition of kidney function. If a sustained decrease in urinary 3-MH is observed through dynamically monitor, it might demonstrate that the progression of renal injury which could provide an early warning for the continuous deterioration of renal function. Additionally, Pfortmueller et al*.* found that the KDIGO guidelines failed to evaluate disease severity for patients with AKI [63]. Nevertheless, the nomogram score composed of urinary 3-MH could assist severity assessment. For SA-AKI patients with higher scores, early initiation of treatment such as renal replacement therapy (RRT) could be considered to avoid prolonged renal damage and improve survival [64]. In addition to its application in SA-AKI, urinary 3-MH could also be employed as a screening instrument for sepsis. Although SCr failed to distinguish between healthy volunteers and sepsis patients, urinary 3-MH exhibits significant differences in our clinical cohort, suggesting its potential as an effective biomarker for sepsis screening. Furthermore, the acquisition of urine samples is non-invasive [65] and urinary 3-MH could be detected quantitatively [66], which could facilitate initial identification of renal injury and promote the precision management of patients with sepsis.

However, decreased biomarkers in septic states are inherently more challenging to interpret than those increased, such as kidney injury molecule-1 (KIM-1), neutrophil gelatinase-associated lipocalin (NGAL), etc. These markers mainly indicate the damage to renal tubules [67, 68]. Nevertheless, renal 3-MH exhibits advantages for filtration functional assessment through its dynamic relationship with GFR. We have found that changes in GFR occur earlier than those increased biomarkers [69]. Furthermore, urinary 3-MH could not only reflect glomerular filtration function, but also is associated with the reabsorption processes. The dual assessment characteristics establish urinary 3-MH as a promising functional biomarker for SA-AKI. On the other hand, decreased biomarkers are more tendency to appear false results when detected using routine clinical methods [70–72]. However, urinary metabolites could be quantitatively detected via Liquid Chromatography-Tandem Mass Spectrometry (LC–MS/MS) technology [66], which has stable repeatability to ensure consistent results and high sensitivity to capture the alterations of metabolites at low concentrations [73]. The established threshold of 3-MH for SA-AKI was 8.68 log_2_(ng/ml), which is valuable to enable precise clinical diagnosis. Furthermore, previous studies demonstrated that the blood 3-MH level increased during renal function impairment [74, 75], contrary to the decreasing trend observed in urinary 3-MH. In light of these findings, we hypothesis that blood-to-urine 3-MH ratio could significantly improve the detection sensitivity of renal injury and represent as a robust diagnostic biomarker for SA-AKI.

There are several limitations to this study. Firstly, SA-AKI mice were only established by intraperitoneal (*i.p.*) injection of LPS. The cecal ligation and puncture (CLP) mice model could simulate the dynamic change process of the immune response in patients with sepsis, while the LPS mice model only focuses on systemic inflammation [76, 77]. However, some factors leading to the instability of SA-AKI model appeared in the process of CLP modeling, such as the length of cecal ligation or the amount of fecal extrusion, which could affect the stability of CLP-induced SA-AKI mice. Therefore, the LPS-induced SA-AKI mice was employed, but a series of measures were adopted to ensure its accuracy and sensitivity. GFR measurement combined with renal functional indicators such as BUN, KIM-1 was implemented to reflect renal function dynamically [69], which provided a reliable foundation for relevance between the LPS-induced AKI mice experiment and clinical translational biomarkers. Furthermore, we plan to optimize the CLP modeling protocol to address stability challenges and validate urinary 3-MH using CLP-induced SA-AKI mice.

Secondly, urine output criteria were not adopted to diagnose AKI in our clinical validation cohort, which may result in an underestimation of the true incidence of AKI. Nevertheless, diagnosing AKI only based on SCr levels reflects a real-world situation of AKI and sepsis [78, 79], particularly given the challenges of accurately monitoring hourly urine output in our ICU due to heavy medical burden and limited staffing. Thirdly, the characteristics of septic patients enrolled in the single—center were difficult to represent the overall population. Patients with AKI were merely caused by sepsis, which induced diversity decreasing. Lifestyle factors such as medication might also limit the generalizability of urinary 3-MH. Moreover, the delay in recruiting patients may affect the tendency alteration observed in the KDIGO phase I to III stages. To overcome these clinical limitations, we propose a multicenter prospective cohort study, through recruiting a more diverse patient population, establishing standardized measurement protocols for serum and urinary 3-MH levels at admission and conducting rigorous follow-up assessments for AKI development to systematically enhance the clinical applicability and generalizability of urinary 3-Methylhistidine.

## Conclusions

3-Methylhistidine (3-MH) was identified as a potential biomarker through renal and urinary metabolomics. Urinary quantitative metabolomic analysis of a clinical cohort was performed to develop diagnostic model based on 3-MH and clinical variables, enabling the detection of SA-AKI. These results are still preliminary findings, further prospective studies in diverse clinical population cohorts are required to determine the accuracy and reliability of 3-methylhistidine.

## Supplementary Information

Additional file 1

Additional file 2

Additional file 3

Additional file 4

Additional file 5

Additional file 6

Additional file 7

Additional file 8

Additional file 9

Additional file 10

Additional file 11

Additional file 12

## Data Availability

All data and analysis methods were described in the article. Renal and urinary metabolomics, renal spatiotemporal metabolomics of 3-Methylhistidineand targeted quantitative urinary 3-Methylhistidine data are available in the Additional files.

## Abbreviations

SA-AKI: Sepsis-associated acute renal injury
3-MH: 3-Methylhistidine
ICU: Intensive care unit
GFR: Glomerular filtration rate
BUN: Blood urea nitrogen
SCr: Serum creatinine
KDIGO: Kidney disease improving global outcomes
NGAL: Neutrophil gelatinase-associated lipocalin
KIM-1: Kidney injury molecule-1
L-FABP: Liver-type fatty acid binding protein
AFP: Alpha-fetoprotein protein
HPLC–MS: High performance liquid chromatography-mass spectrometry
NMR: Nuclear magnetic resonance
LR: Logistic regression
CKD: Chronic kidney disease
SD: Standard deviation
OPLS-DA: Orthogonal partial least squares-discriminant analysis
VIP: Variable importance in the projection
APACHE II: Acute physiological and chronic health assessment
SOFA: Sequential organ failure assessment
RRT: Renal replacement therapy
MSI: Mass spectrometry image
UPLC-MS/MS: Ultra performance liquid chromatography mass spectrometry
LC–MS/MS: Liquid chromatography–tandem mass spectrometry
CLP: Cecal ligation and puncture
FC: Fold change
OR: Odds ratio
ROC: Receiver operating characteristic
