# Supplementary material for: Urinary 3-methylhistidine as a potential biomarker for sepsis-associated acute kidney injury: multidimensional metabolomics analysis in mice and human
Source: Ann Intensive Care. 2025 Aug 26;15:125. doi: 10.1186/s13613-025-01550-z (PMC12380662; doi:10.1186/s13613-025-01550-z)
Supplement: Supplementary file 1 — Additional file 1 [file 13613_2025_1550_MOESM1_ESM.docx]

**SA-AKI mice Construct**

At 0h, 8h, and 24h post-PBS or LPS intervention, mice in each group were anesthetized with isoflurane using a small animal anesthesia machine (Surgivet, USA) and sacrificed via cervical dislocation.

The abdominal wall of the mice was cut open along the midline, the renal was removed and trimmed of unnecessary tissue, and then the renal tissues were placed in sterile cryotubes and stored in liquid nitrogen for renal sequencing. The collected blood samples were centrifuged at 3000g for 15 minutes, after which the upper layer of plasma was extracted for blood urea nitrogen testing.

Urine samples were collected from each mouse in the second part. Twenty-four hours post LPS or PBS intervention, mild pressure was applied in the lower abdomen of mice to induce urine excretion. The urine samples were collected and centrifuged at 4,000×g for 10 minutes at 4°C, and supernatants were stored at -80°C for urinary metabolomics sequencing.

**Real-time fluorescence imaging technology**

The right abdominal hair of the mice was shaved, and a depilatory cream was subsequently used to clean the right abdominal area. The mice were anesthetized with isoflurane, and the optical device (from MediBeacon GmbH, Mannheim, Germany) was fixed to the shaved area. Once the mice regained consciousness, they were intravenously injected with mice sinistrin via the tail vein within 5 minutes to monitor fluorescence intensity, which was continuously observed for 1 hour. After removing the device under isoflurane anesthesia, the data was analyzed using MB Studio v. 22 (Medibeacon GmbH). During the monitoring process, the mice could move freely, with unrestricted access to water and food.

**Mice Biomarker Testing**

Urine was used for KIM-1 testing. A reagent kit (E-EL-M3039) was used to measure KIM-1 according to the manufacturer's instructions (Elabscience, Wuhan, China.). An automatic biochemical analyzer (IDEEX) was used to determine blood urea nitrogen.

**Targeted Quantitative Metabolomics for Clinical Urine Samples.**

In our study, Liquid Chromatography-Tandem Mass Spectrometry (LC-MS/MS) (25) was employed on urine samples from healthy volunteers and patients with sepsis or sepsis-associated acute kidney injury (SA-AKI) to detect 3-Methyhistidine (3-MH).

**1. Sample Processing and Isolation**

Urine samples were retrieved from the -80°C freezer. After thawing, the urine samples were vortexed separately for 10 seconds. A 50 µL aliquot of each sample was transferred into a centrifuge tube and mixed with 250 µL of 20% acetonitrile/methanol solution. The mixture was vortexed for 3 minutes and centrifuged at 12,000 r/min for 10 minutes at 4°C. Subsequently, 250 µL of the supernatant was transferred to a new centrifuge tube and stored at -20°C for 30 minutes. The supernatant was then centrifuged again at 12,000 r/min for 10 minutes at 4°C. Finally, 180 µL of the supernatant was passed through a protein precipitation plate for subsequent LC-MS analysis.

**2. UPLC Operating Conditions**

**2.1. T3 method**

For the T3 method, chromatographic separation was achieved using a Waters ACQUITY UPLC HSS T3 C18 column (100 mm × 2.1 mm, 1.8 µm). The mobile phase consisted of 0.05% formic acid in water (Phase A) and 0.05% formic acid in acetonitrile (Phase B)(26). A gradient elution program was employed, beginning at 5% B at time 0, increasing to 95% B between 8 to 9.5 minutes, and then returning to 5% B from 9.6 to 12 minutes. The flow rate was maintained at 0.35 mL/min, with the column temperature set at 40°C. The injection volume for each run was 2 µL.

**2.2. Amide method**

Chromatographic separation for the amide method was performed using an ACQUITY UPLC BEH Amide column (2.1 × 100 mm, 1.7 μm). The solvent system consisted of water with 10 mM ammonium acetate and 0.3% ammonium hydroxide (Phase A) and 90% acetonitrile/water (V/V) (Phase B)(). The gradient started at 95% B from 0 to 1.2 minutes, then decreased to 70% B at 8 minutes, followed by a further decrease to 50% B between 9 to 11 minutes. Finally, the gradient returned to 95% B from 11.1 to 15 minutes. The flow rate was set at 0.4 mL/min, with the column temperature maintained at 40°C, and an injection volume of 2 μL was used for each analysis.

**3. ESI-MS/MS operating Conditions**

Linear ion trap (LIT) and triple quadrupole (QQQ) scans were conducted using a QTRAP® 6500+ LC-MS/MS system (Sciex) equipped with an ESI Turbo IonSpray interface. The system operated in both positive and negative ion modes and was controlled by Analyst 1.6.3 software (Sciex). The ESI source parameters were set as follows: the ion source was configured for ESI+/- mode, with a source temperature of 550°C, ion spray voltage of 5500 V for positive ion mode and -4500 V for negative ion mode, and the curtain gas (CUR) was maintained at 35 psi. Metabolite analysis was performed using scheduled multiple reaction monitoring (MRM), with data acquisition carried out using Analyst 1.6.3 software (28). Metabolite quantification was completed with Multiquant 3.0.3 software (Sciex). The mass spectrometer parameters, including declustering potentials (DP) and collision energies (CE), were optimized for each individual MRM transition. Specific sets of MRM transitions were monitored during each elution period based on the metabolites present in that time frame.

**4. Metabolites detection system**

3-Methyhistidine detected by MetWare (<http://www.metware.cn/>) using the AB Sciex QTRAP 6500 LC-MS/MS platform.

**Untargeted Spatial Metabolomics Sequencing on Renal Tissue**

**1. Sample Preparation and Sectioning**

Frozen kidney tissue samples were fixed with three drops of distilled water during the cutting stage. The tissues were then sectioned into 10 μm thick slices using a Leica CM1950 cryostat (Leica Microsystems GmbH, Wetzlar, Germany) at -20°C (29). Following sectioning, the tissue slices were placed on electrically conductive slides coated with indium tin oxide (ITO) and dried for 30 minutes in a vacuum desiccator.

**2. Matrix Application**

The dried tissue sections mounted on ITO glass slides were coated using an HTX TM sprayer (Bruker Daltonics, Germany) with a solution of 15 mg/mL DHB (2,5-dihydroxybenzoic acid) in a 90:10 acetonitrile mixture (30). The sprayer was set to a temperature of 60°C, with a flow rate of 0.12 mL/min and a pressure of 6 psi. Matrix application involved 28 passes over the slides, with 5 seconds of drying time between each pass.

**3. Mass Spectrometry Imaging (MSI)**

MALDI timsTOF MSI experiments were conducted using a prototype Bruker timsTOF flex MS system (Bruker Daltonics, Bremen, Germany) equipped with a 10 kHz smartbeam 3D laser. The laser power was maintained at 90% throughout the experiment. Mass spectra were recorded in positive ion mode over a mass range of m/z 50-1300 Da (31). The spatial resolution for tissue imaging was set to 20 μm, with each spectrum derived from 400 laser shots. The MALDI mass spectra were normalized using the Root Mean Square method, and signal intensities were displayed as normalized intensities (32). Further structural verification of identified metabolites was achieved through MS/MS fragmentations performed on the timsTOF flex MS system in MS/MS mode.

**Ultra-performance liquid chromatography(UPLC/MS) for Urine Samples**

**1.Sample Preparation and Extraction**

**1.1. Extraction Methods for Hydrophilic Compounds**

Liquid Samples (Class I): Samples stored at -80°C were thawed on ice and vortexed for 10 seconds. 50 μL of sample and 300 μL of extraction solution (ACN= 1:4, V/V) containing internal standards were added to a 2 mL microcentrifuge tube. The mixture was vortexed for 3 minutes, then centrifuged at 12,000 rpm for 10 minutes at 4°C. 200 μL of the supernatant was collected and stored at -20°C for 30 minutes, then centrifuged again at 12,000 rpm for 3 minutes at 4°C. Finally, 180 μL of the supernatant was transferred for LC-MS analysis (33).

**1.2. Extraction Methods for Hydrophobic Compounds**

Liquid Samples (Class I): Samples were removed from -80°C storage, thawed on ice, and vortexed for 10 seconds. 50 μL of the sample was mixed with 1 mL of extraction solvent (MTBE= 3:1, V/V) containing internal standards. The mixture was vortexed for 15 minutes, followed by the addition of 200 μL of water. After vortexing for 1 minute, the sample was centrifuged at 12,000 rpm for 10 minutes. 200 μL of the upper organic layer was collected and evaporated using a vacuum concentrator (34). The dried extract was reconstituted in 200 μL of solution (ACN= 1:1, V/V) for LC-MS/MS analysis.

**2. UPLC Conditions**

**2.1. UPLC Conditions for Hydrophilic Compounds**

Sample extracts were analyzed using an LC-ESI-MS/MS system (UPLC, ExionLC AD; MS, QTRAP® System). UPLC conditions: column: Waters ACQUITY UPLC HSS T3 C18 (1.8 μm, 2.1 mm*100 mm); column temperature: 40°C; flow rate: 0.4 mL/min; injection volume: 2 μL; solvent system: water (0.1% formic acid) (0.1% formic acid) (35); gradient program: 95:5 V/V at 0 min, 10:90 V/V at 11.0 min, 10:90 V/V at 12.0 min, 95:5 V/V at 12.1 min, 95:5 V/V at 14.0 min.

**2.2. UPLC Conditions for Hydrophobic Compounds**

Sample extracts were analyzed using an LC-ESI-MS/MS system (UPLC, ExionLC AD; MS, QTRAP® System). UPLC conditions: column: Thermo Accucore™ C30 (2.6 μm, 2.1 mm*100 mm i.d.); solvent system: A: acetonitrile/water (60/40, V/V, 0.1% formic acid, 10 mmol/L ammonium formate), B: acetonitrile/isopropanol (10/90 V/V, 0.1% formic acid, 10 mmol/L ammonium formate); gradient program: A/B at 80:20 V/V at 0 min, 70:30 V/V at 2.0 min, 40:60 V/V at 4 min, 15:85 V/V at 9 min, 10:90 V/V at 14 min, 5:95 V/V at 15.5 min, 5:95 V/V at 17.3 min, 80:20 V/V at 17.3 min, 80:20 V/V at 20 min; flow rate: 0.35 mL/min; temperature: 45°C; injection volume: 2 μL. The effluent was connected to an ESI-triple quadrupole-linear ion trap (QTRAP)-MS.

**3. QTOF-MS/MS**

The Triple TOF mass spectrometer was used for MS/MS spectrum acquisition in information-dependent acquisition (IDA) mode during LC/MS analysis. The software (TripleTOF 6600, AB SCIEX) continuously evaluates full scan MS data and triggers MS/MS acquisition based on predefined criteria. In each cycle, 12 precursor ions with intensity greater than 100 are selected for fragmentation at a collision energy (CE) of 30 V (12 MS/MS events with 50 ms accumulation time each) (36). ESI source conditions: ion source gas 1 set at 50 psi, ion source gas 2 at 50 psi, curtain gas at 25 psi, source temperature at 500°C, and ion spray voltage floating (ISVF) at 5500 V or -4500 V for positive and negative modes, respectively.

**4. ESI-Q TRAP-MS/MS**

**4.1. ESI-Q TRAP-MS/MS for Hydrophilic Compounds**

LIT and triple quadrupole (QQQ) scans were performed using a triple quadrupole-linear ion trap mass spectrometer (QTRAP), specifically the QTRAP® LC-MS/MS system. This system was equipped with an ESI Turbo Ion-Spray interface and operated in both positive and negative ion modes, controlled by Analyst 1.6.3 software (Sciex). The operating conditions for the ESI source were: source temperature at 500°C, ion spray voltage at 5500 V for positive mode and -4500 V for negative mode, with ion source gases I (GSI) and II (GSII) set to 55 and 60 psi, respectively, and curtain gas (CUR) set to 25 psi. The collision gas (CAD) was set to high (37). Instrument tuning and mass calibration were performed using 10 and 100 μmol/L polypropylene glycol solutions in QQQ and LIT modes, respectively. A specific set of MRM transitions was monitored for each time segment based on the metabolites eluting during that period.

**4.2. ESI-Q TRAP-MS/MS for Hydrophobic Compounds**

LIT and triple quadrupole (QQQ) scans were acquired on a triple quadrupole-linear ion trap mass spectrometer (QTRAP), utilizing the QTRAP® LC-MS/MS system. This system was equipped with an ESI Turbo Ion-Spray interface and operated in positive and negative ion modes, controlled by Analyst 1.6.3 software (Sciex). The ESI source settings were as follows: source temperature at 500°C, ion spray voltage at 5500 V for positive mode and -4500 V for negative mode. Ion source gas 1 (GS1) was set to 45 psi, gas 2 (GS2) to 55 psi, and curtain gas (CUR) to 35 psi, with the collision gas (CAD) set to medium. Instrument tuning and mass calibration were conducted with 10 and 100 μmol/L polypropylene glycol solutions in QQQ and LIT modes, respectively. QQQ scans were performed as MRM experiments, with the collision gas (nitrogen) set to 5 psi (38). The declustering potential (DP) and collision energy (CE) for individual MRM transitions were further optimized. A specific set of MRM transitions was monitored for each time period, corresponding to the metabolites eluting during that phase.
