# Supplementary figures and images for: Urinary 3-methylhistidine as a potential biomarker for sepsis-associated acute kidney injury: multidimensional metabolomics analysis in mice and human
Source: Ann Intensive Care. 2025 Aug 26;15:125. doi: 10.1186/s13613-025-01550-z (PMC12380662; doi:10.1186/s13613-025-01550-z)

KIM-1

20

10

0

\*\*

Control

SA-AKI

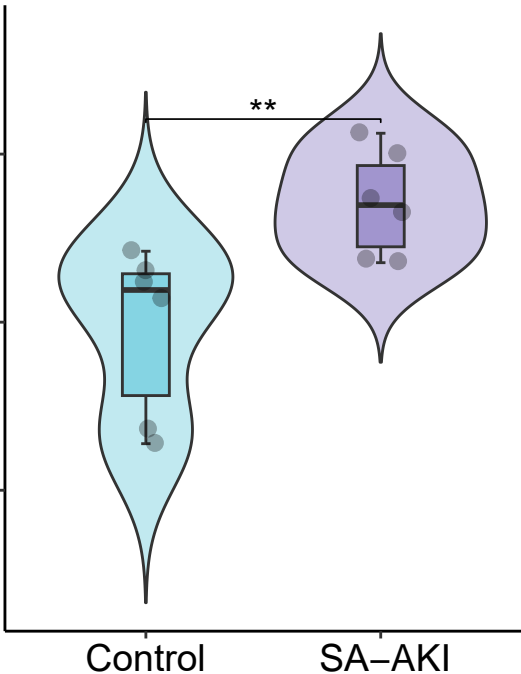

Supplement: Supplementary file 2 — Additional file 2 [file 13613_2025_1550_MOESM2_ESM.pdf]

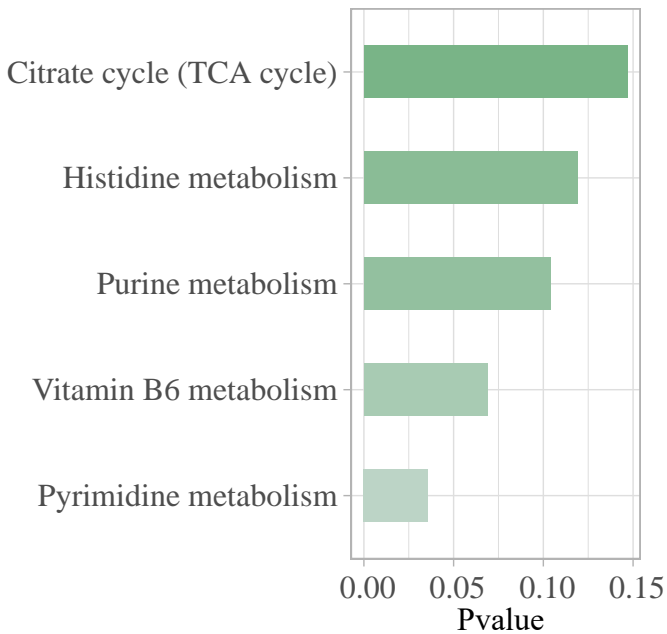

Supplement: Supplementary file 3 — Additional file 3 [file 13613_2025_1550_MOESM3_ESM.pdf]

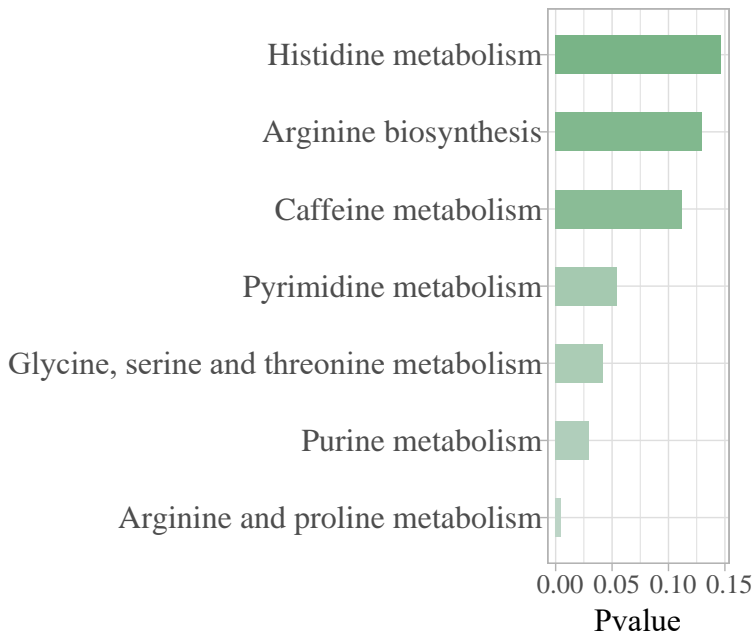

Supplement: Supplementary file 4 — Additional file 4 [file 13613_2025_1550_MOESM4_ESM.pdf]

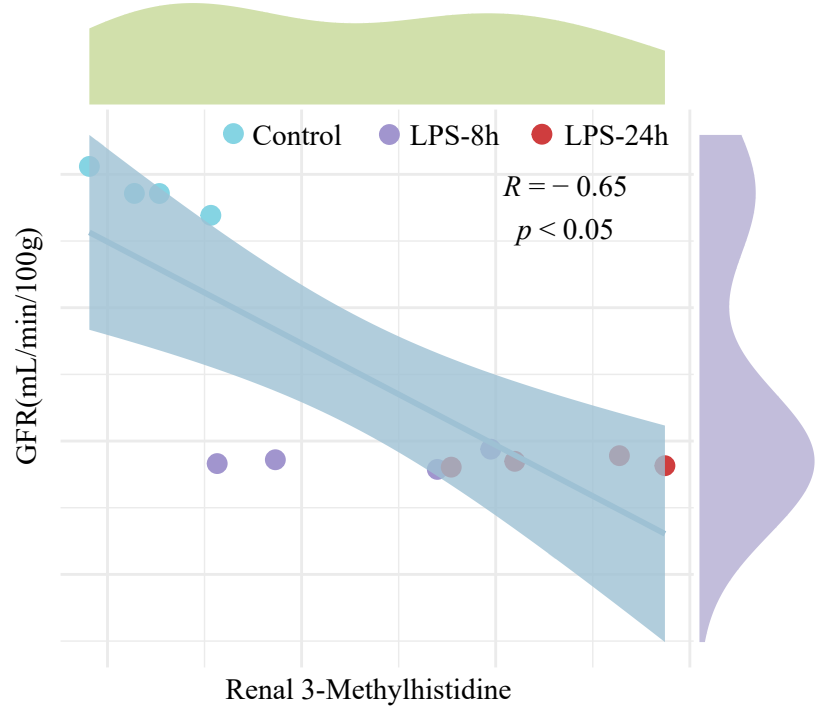

Supplement: Supplementary file 5 — Additional file 5 [file 13613_2025_1550_MOESM5_ESM.pdf]

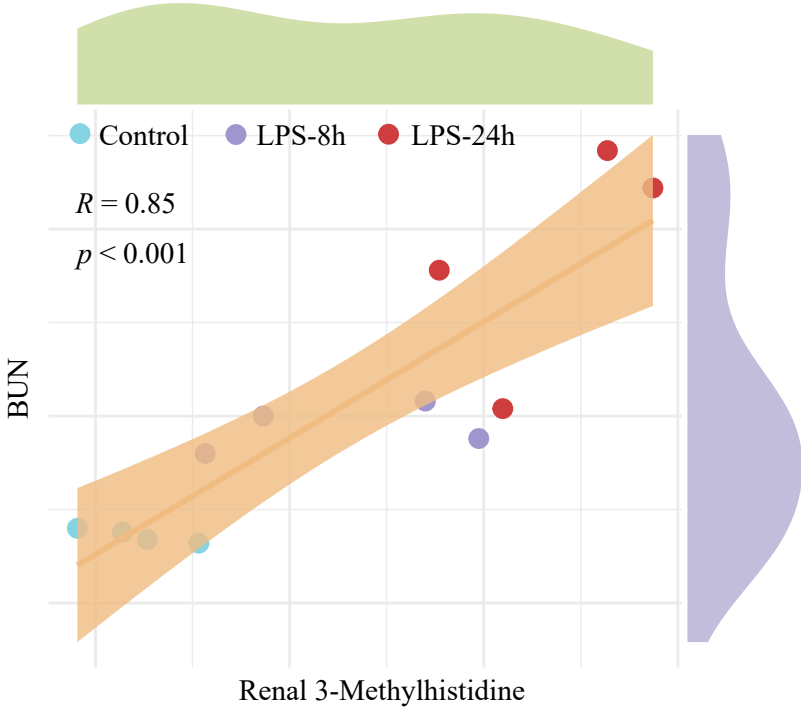

Supplement: Supplementary file 6 — Additional file 6 [file 13613_2025_1550_MOESM6_ESM.pdf]

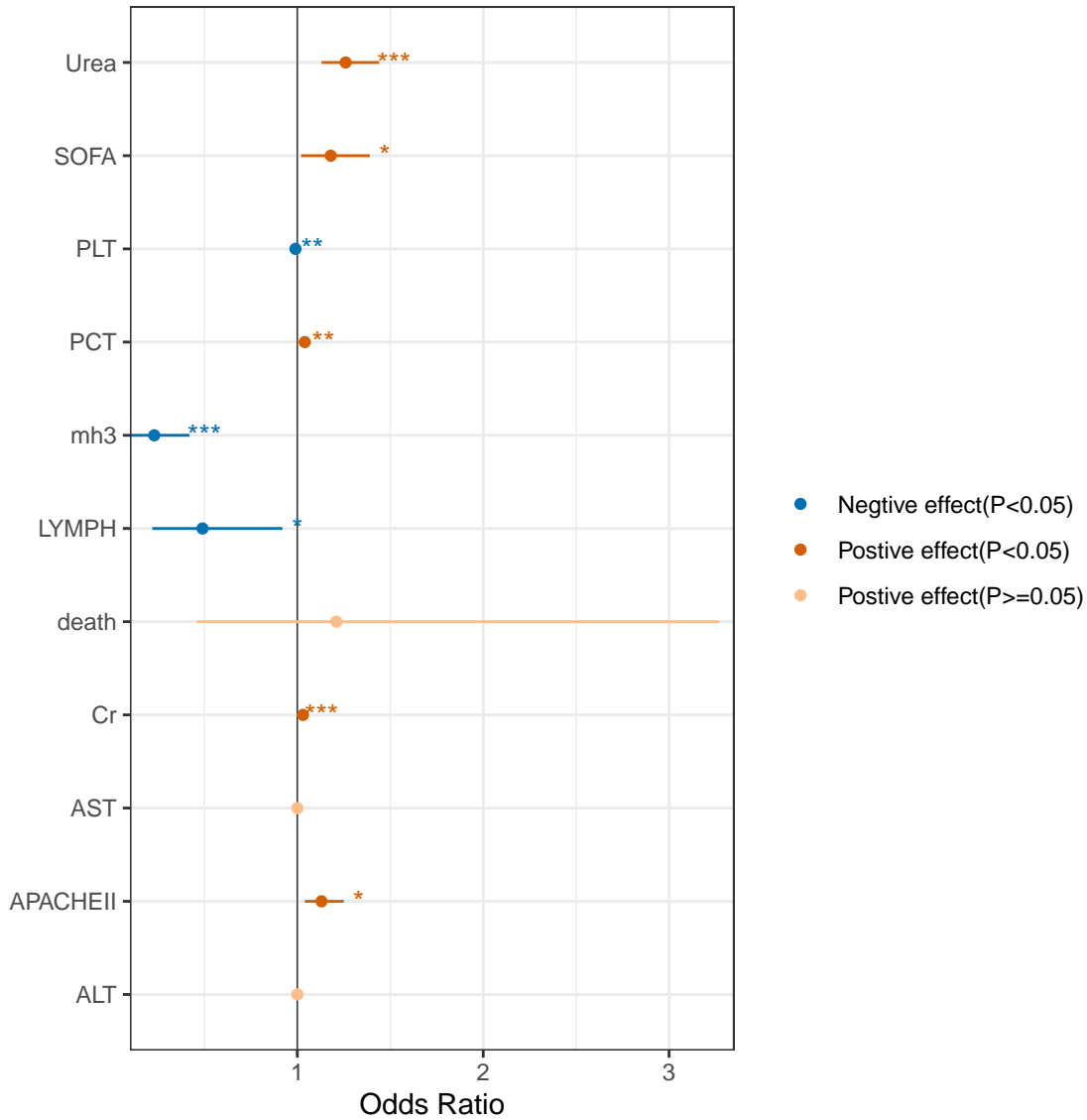

Supplement: Supplementary file 7 — Additional file 7 [file 13613_2025_1550_MOESM7_ESM.pdf]

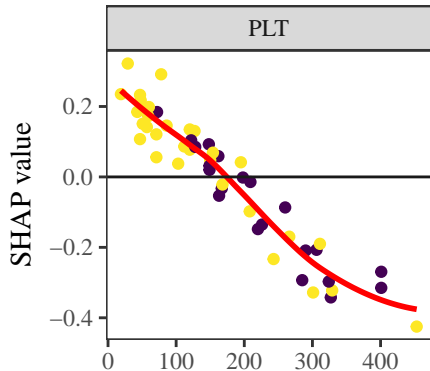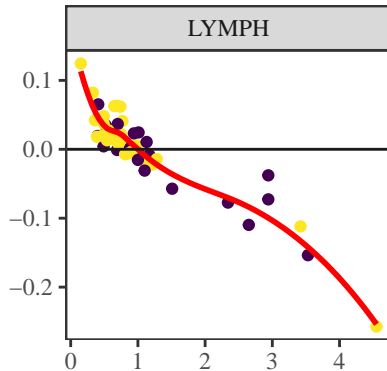

● Sepsis ● SA-AKI

Supplement: Supplementary file 8 — Additional file 8 [file 13613_2025_1550_MOESM8_ESM.pdf]
